# Spontaneous Middle Cranial Fossa Cerebrospinal Fluid Leaks and Intracranial Hypertension: Systematic Review With Meta‐Analysis

**DOI:** 10.1002/lio2.70439

**Published:** 2026-05-12

**Authors:** Georgios Vavoulis, Dimitrios Spinos, Panagiotis Varoutis, Georgios Georgountzos, Nina Rafailia Karela, Georgios Geropoulos, Jameel Muzaffar, Wai Sum Cho

**Affiliations:** ^1^ Department of Neurosurgery General Attica Hospital “KAT” Kifissia Greece; ^2^ Department of Cancer and Genomics University of Birmingham Birmingham UK; ^3^ Department of Otolaryngology, Head and Neck Surgery University Hospitals Birmingham NHS Foundation Trust Birmingham UK; ^4^ Department of Neurosurgery Ippokrateio General Hospital of Thessaloniki Thessaloniki Greece; ^5^ Department of Neurosurgery General Hospital of Nikaia “Agios Panteleimon” Nikaia Greece; ^6^ School of Biomedical Engineering and Imaging Sciences King's College London London UK; ^7^ Department of Biomedical Engineering University of West Attica Aigaleo Greece; ^8^ School of Medicine National and Kapodistrian University of Athens Athens Greece; ^9^ West Hertfordshire Teaching Hospitals Watford UK; ^10^ Department of Otolaryngology, Head and Neck Surgery Nottingham University Hospitals NHS Trust Nottingham UK

**Keywords:** middle cranial fossa, shunt, spontaneous cerebrospinal fluid leak, systematic review, temporal bone

## Abstract

**Introduction:**

The perioperative management of spontaneous cerebrospinal fluid leaks (sCSFL) of lateral skull base remains under debate. We systematically synthesized and evaluated the current literature, to investigate the perioperative cranial‐pressure assessment protocols and relief methods. The assessment corresponded to fulfillment of criteria for idiopathic intracranial hypertension (IIH) or otherwise defined in the study. Relief methods included either medical, that is, acetazolamide, or invasive, that is, lumbar puncture (LP) or drain (LD) or ventriculoperitoneal shunt (VPS).

**Methods:**

Selected studies concerned surgical treatment of lateral sCSFL. Data extracted included the following: study characteristics, patient characteristics, primary outcomes, and secondary outcomes. Data sources: MEDLINE, EMBASE, and Cochrane Library.

**Results:**

Baseline data were calculated from 2039 operations, out of which 423 had perioperative LP or LD. When only studies which mentioned recurrence or persistence rates were considered, 262 of them were used routinely or in selected patients and 117 occasionally. In those two cohorts, recurrence or persistence rates were similar (10.1% vs. 8.9%). Intracranial pressure was assessed in 335 patients from 10 studies and 48 among them were diagnosed with IIH (14.3%). Acetazolamide was inconsistently used in 17.7% of cases. From 18 studies that reported the use of VPS, this was used in 11.2% of the cases.

**Discussion:**

Systematic perioperative LP or LD use does not affect the recurrence or persistence rates. However, decision making with respect to the use of LP or LD is inconsistently reported in the literature. The same issue holds for the use of acetazolamide or VPS. Assessment of intracranial hypertension is usually neglected, and when used, not standardized.

**Level of Evidence:**

2

## 1 Introduction

Spontaneous cerebrospinal fluid leaks (sCSFL) of the temporal bone are increasingly recognized in clinical practice [1, 2, 3]. The principal risk associated with these defects is the potential development of meningitis or other serious intracranial infectious complications, making definitive surgical closure essential. Unlike leaks arising from trauma or iatrogenic injury, sCSFLs are strongly correlated with underlying elevated intracranial pressure (ICP), often linked to idiopathic intracranial hypertension (IIH) [2] and further correlated to obstructive sleep apnea (OSA) [4]. This patient cohort typically consists of obese, middle‐aged women [5, 6, 7]. Chronically increased ICP is believed to cause progressive thinning and attenuation of the skull base, particularly the tegmen tympani and mastoideum, ultimately leading to dural dehiscence and fistula formation [3, 8, 9, 10]. A significant challenge in managing these patients is the difficulty in diagnosing the underlying IIH. The active CSF leak often acts as a “pressure release valve,” potentially normalizing ICP and thereby masking the typical clinical signs (such as papilledema or chronic headache) and leading to falsely low lumbar puncture (LP) opening pressures (OPs) [7, 10]. Although surgical repair strategies utilizing approaches like transmastoid (TM) or middle cranial fossa (MCF) yield high initial success rates (frequently reported between 93% and 96%) [1, 5, 9, 11], surgical closure alone may be inadequate for long‐term resolution [9, 12]. Untreated elevated ICP is a primary predictor of recurrence, metachronous leaks at new sites, and eventual surgical failure [2, 3, 9]. Consequently, effective, long‐term management requires addressing the underlying ICP dysregulation through various adjunctive measures. These management strategies are often individualized, contributing to variability in reported outcomes. Temporary CSF diversion is frequently achieved using a lumbar drain (LD), which may be placed preoperatively or intraoperatively to facilitate temporal lobe relaxation and left postoperatively for 48–72 h to reduce pressure on the repair site [2, 9, 13]. Acetazolamide is used, mostly postoperatively, to decrease CSF production in patients with elevated ICP [5, 12, 14, 15]. Finally, long‐term surgical CSF diversion, typically via VPS, is reserved for patients with refractory IIH, high ICP, high‐risk characteristics (e.g., high BMI, empty sella), or recurrence despite primary repair [6, 9, 12, 13]. The optimal timing and specific criteria for initiating these adjunctive ICP management interventions—ranging from temporary LD usage to permanent shunting—remain points of debate in the literature. This paper aims to analyze the evidence concerning the identification of IIH and the roles of LD, acetazolamide and CSF shunting in optimizing the clinical outcomes and reducing recurrence risk for patients with sCSFL.

## 2 Materials and Methods

### 2.1 Study Design

This study was performed according to the Preferred Reporting Items for Systematic Reviews and Meta‐analyses (PRISMA) guidelines for systematic reviews [16]. The protocol was registered in PROSPERO (International Prospective Register of Systematic Reviews) on June 21, 2022 (reference code: CRD42022340068). Selection criteria were structured according to the PICO framework as follows:

– P (participants): Patients of any age and sex with sCSFL of MCF that underwent surgery.
– I (intervention): Perioperative use of LP or LD or VPS.
– C (comparison): Systematic and routine perioperative use of LD for surgical sCSFL patients was compared with occasional perioperative use.
– O (outcomes): Primary outcomes were recurrence or persistence rates. Secondary outcomes were successful management of recurrences or persistent leaks with cranial pressure relief methods and their complications.

Original clinical studies, published in English, investigating the spontaneous CSF leak of MCF were included.

Exclusion criteria were the following:

- Articles reporting only nonspontaneous/acquired CSF leaks.
- Articles published in a language other than English.
- Narrative or systematic reviews and meta‐ analyses.
- Animal and in vitro studies.
- Case reports, errata, comments, perspectives, letters to the editor, and editorials that did not provide any primary patient data.
- Published abstracts with no available full text.
- Case series with less than five patients.
- Irrelevant studies (irrelevant population: anterior or posterior skull base CSF leaks, patients with previous history of trauma, neoplasm, brain or skull base surgery or active chronic otitis media).

### 2.2 Search Strategy

Databases searched: MEDLINE via PubMed, EMBASE via Ovid, and Cochrane Library via Central databases were searched during the selection process (end‐of‐search date: April 2025). The search strategy was developed with the guidance of a Clinical Librarian and included the following search terms: (((((csf) OR ((cerebrospinal) AND (fluid))) AND (leak)) AND (spontaneous)) OR (meningocele) OR (encephalocele) OR (meningoencephalocele) OR ((spontaneous) AND (otorrhea))) AND (((lateral) AND (skull) AND (base)) OR ((temporal) AND (bone)) OR ((middle) AND (cranial) AND (fossa))). Two reviewers independently screened abstracts and subsequently full texts using the online COVIDENCE platform (Covidence systematic review software, Veritas Health Innovation, Melbourne, Australia; available at www.covidence.org). Disagreements were resolved by discussion, and if required arbitration by a third reviewer.

### 2.3 Data Extraction

Two reviewers independently extracted relevant data through a standardized data extraction template. Any conflicts were resolved through discussion and consultation with a third reviewer. Data extracted included the following: (i) study characteristics (first author, year of publication, study design, and number of patients); (ii) patient characteristics (age, body mass index [BMI], sex, follow‐up and revision surgeries); (iii) the use of temporary (LD) or permanent (shunt) CSF diversion method; (iv) primary outcome (recurrence); and (v) secondary outcome (complications).

### 2.4 Quality of Evidence Assessment

Quality of included studies was assessed employing the National Heart, Lung and Blood Institute (NHLB) Assessment Tool for case–control studies (12 criteria), observational cohort and cross‐sectional studies (14 criteria), and case series studies (9 criteria). Studies were classified as poor, fair, or good quality for each item of the assessment tool. There is no specific score threshold that would categorize a study as poor, fair, or good; thus, the assessment was subjective, and any discrepancies were resolved by a third reviewer. Before the quality assessment stage, it was decided that “poor” studies would be excluded.

### 2.5 Statistical Analysis

Obtainable data from included studies regarding approach‐based outcomes were further investigated through a single‐arm proportional analysis. Statistical analysis was performed among studies reporting outcomes (recurrence or persistence rates) for perioperative use of LP with or without LD. Statistics were analyzed with R (version 4.4.2, R Foundation for Statistical Computing). A single‐arm proportional analysis with 95% confidence intervals was used. A random effects model with logit transformation was utilized since the main proportion was < 0.2 and not equal to zero. The inverse variance method was applied to account for individual study weights. When the number of analyzed studies was ≥ 10, publication bias was assessed through funnel plot and rank correlation test for funnel plot asymmetry, whereas heterogeneity among included studies was evaluated with I2 and Baujat plots.

## 3 Results

### 3.1 Study Selection and Characteristics

**FIGURE 1:**
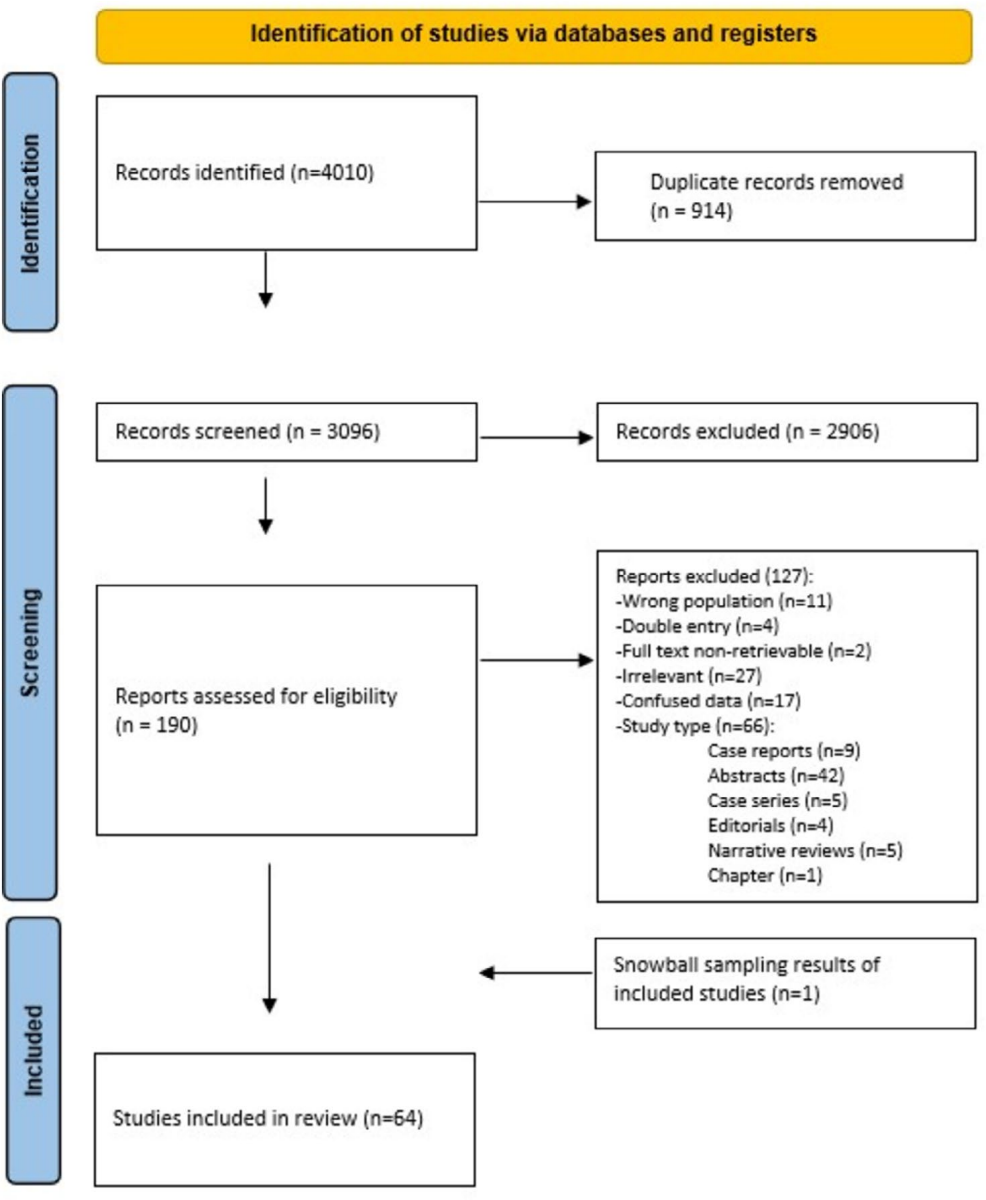
PRISMA flowchart.

A systematic search identified 4010 abstracts. After the removal of duplicates and title/abstract review, 190 articles underwent full‐text review. Of these, 63 articles focus on the surgical treatment of sCSFL patients and commented on IIH investigation or perioperative use of LP, LD, acetazolamide or VPS. One article was retrieved via snowball sampling. This process is described in more detail in the PRISMA flowsheet (Figure 1).

### 3.2 Baseline Characteristics

This review included 2039 sCSFL operations, with a female rate of 52.6% (1073), mean age 55.8 (SD 5.5) years and mean BMI 34.5 (SD 3.3). Preoperatively, 318 had LP or LD and postoperatively 114. Persistence or recurrence was reported for 1640 patients and 128 of them had either one (7.8%). Mean follow up was 23.1 (SD 16.8) months [1, 2, 3, 5, 6, 7, 8, 9, 10, 11, 12, 13, 14, 15, 17, 18, 19, 20, 21, 22, 23, 24, 25, 26, 27, 28, 29, 30, 31, 32, 33, 34, 35, 36, 37, 38, 39, 40, 41, 42, 43, 44, 45, 46, 47, 48, 49, 50, 51, 52, 53, 54, 55, 56, 57, 58, 59, 60, 61, 62, 63].

### 3.3 Lumbar Puncture and Drain

Perioperative LP with or without LD placement (LP/LD) was rarely used routinely and indications were loose (Table 1). Some authors supported their use in patients that were deemed high‐risk for recurrence, including those with signs suggestive of IIH [7, 64], history of failed previous surgical repair [8] and inflammatory processes [64]. Some groups described a strategy of placement when identifying intraoperative findings such as profuse leak [8, 18, 64] or multiple defects [64], while there were some groups that relied on surgeon's preference and experience with respect to cranial approach [19, 20]. Routine use of LP/LD was described across high‐risk patients due to their underlying disease [9, 12, 13, 21, 22, 23] or in the case of patients requiring tight postoperative monitoring [13, 24]. Stevens et al. [14] and Son et al. [25], on the other hand, reported institutional protocols that guided the decision‐making process of their use.

**TABLE 1:** Studies with routine or specific indication (Rout/Sel) for LP/LD usage.

Study	Operations	Pre‐op LP/LD	Post‐op LP/LD	R/P
Bidot et al. [7]	36	7. Indication: IIH	2	7
Brainard et al. [24]	9	0	9 (routinely, to study IIH/ICP relation)	0
Cooper et al. [26]	46	43 (MCF 39; PCF 3). Routinely	n/a	n/a
Gonen et al. [18]	9	0	3. Indication: intra‐op findings (large defect size, high flow defect)	0
Kenning et al. [9]	23	21/23. Routinely	1	1
May et al. [21]	15	12. Routinely	n/a	2
Nelson et al. [22]^a^	21	10. Routinely	0	0
Oliaei et al. [19]	15	5. Indication: approach other than TM	0	1
Patel et al. [23]	7	7. Routinely.	0	0
Perez et al. [8]	28	0	15. Indication: profuse intra‐op CSF leak or when surgery was a revision	5
Scullen et al. [17]	8	5. Indication: profuse intra‐op leak or large defect	5	0
Son et al. [25]	33	33. Routinely	n/a	2
Stevens et al. [14]	28	28. Routinely	n/a	1
Vivas et al. [12]	34	29. Routinely	n/a	3
Khanna et al. [13]	79	60. Routinely	“Majority of patients” at 2‐week follow‐up.	1
Swanson et al. [20]	43	6. Indication: suspected intracranial hypertension	0	6
Heman‐Ackah et al. [27]	107	9. Indication: IIH	0	10
Excluding studies that did not report recurrence rates	495	232 (46.8%)	30 (6.1%)	39 (7.9%)
All studies	541	275 (55.5%)	30 (6.1%)	n/a

Abbreviations: MCF, middle cranial fossa; PCF posterior cranial fossa; R/P, recurrence or persistence; TM, transmastoid approach.

^a^ The first decade from [22].

We compared recurrence rates between studies where LP/LD was used either routinely or in selected patients (Rout/Sel‐group) and studies where LP/LD was only used occasionally or retrospectively reported (Occ‐group). The routine‐or‐selection group included 495 operations (from 17 studies) and preoperative LP/LD was used in 232 (46.8%) of them (Table 1), while the occasional group included 1219 operations (from 49 studies) out of which preoperative LP/LD was used in 33 (2.7%) of them (Table 2). Interestingly, there was similar postoperative recurrence or persistence of the leak, respectively, 7.9% vs. 10.1%.

**TABLE 2:** Studies with occasional (Occ) use of LP/LD.

Study	Operations	Pre‐op LP/LD	Post‐op LP/LD	R/P
Ahmed et al. [28]	5	0	0	0
Alwani et al. [29]	27	n/a	0	0
Alwani et al. [1]	45	0	n/a	2
Brenet et al. [11]	44	n/a	n/a	2
Brown et al. [30]	9	n/a	n/a	n/a
Cheng et al. [31]	58	0	2	3
Dai et al. [32]	64	n/a	41	11
El Hadi et al. [33]	13	n/a	n/a	n/a
Goddard et al. [34]	23	0	0	3
Grinblat et al. [35]	61	0	n/a	0
Gubbels et al. [36]	15	14	n/a	1
Hatch et al. [37]	18	n/a	n/a	n/a
Hendriks et al. [38]	90	0	0	8
Hoang et al. [39]	21	n/a	n/a	0
Hwa et al. [40]	43	0	1	3
Kari et al. [41]	33	n/a	n/a	1
Kim et al. [10]	16	0	0	1
Kutz et al. [6]	17	0	0	1
Kutz et al. [5]	55	0	0	5
Lee et al. [42]	30	n/a	n/a	n/a
Leonetti et al. [43]	51	0	0	2
Lundy et al. [44]	11	0	n/a	0
Markou et al. [45]	12	0	n/a	0
Mayeno et al. [46]	6	2	2	0
McNulty et al. [47]	50	0	0	n/a
Mostafa et al. [48]	14	4	2	2
Nahas et al. [49]	15	n/a	0	n/a
Nelson et al. [22]^a^	44	0	5	5
Pappas et al. [50]	12	6	n/a	n/a
Patel et al. [51]	31	12	n/a	1
Pelosi et al. [52]	8	0	7	2
Rao et al. [53]	11	1	0	3
Sanna et al. [54]	33	n/a	n/a	n/a
Schwartz et al. [3]	12	3	n/a	n/a
Stevens et al. [14]	47	0	0	11
Stucken et al. [55]	11	0	0	1
Valtonen et al. [56]	5	0	0	1
Walia et al. [57]	28	0	0	1
Wong et al. [58]	14	0	3	0
Yancey et al. [2]	42	0	0	1
Symms et al. [59]	54	n/a	n/a	4
Miller et al. [60]	92	n/a	n/a	4
Shah et al. [15]	60	0	0	5
Hentati et al. [61]	48	n/a	23	9
Quimby et al. [62]	87	n/a	n/a	n/a
Cumpston et al. [63]	13	0	0	1
Excluding studies that did not report recurrence	1219	33 (2.7%)	84 (6.8%)	94 (10.1%)
All studies	1498	43 (3.5%)	84 (6.8%)	n/a

^a^ The second decade from [22].

**FIGURE 2:**
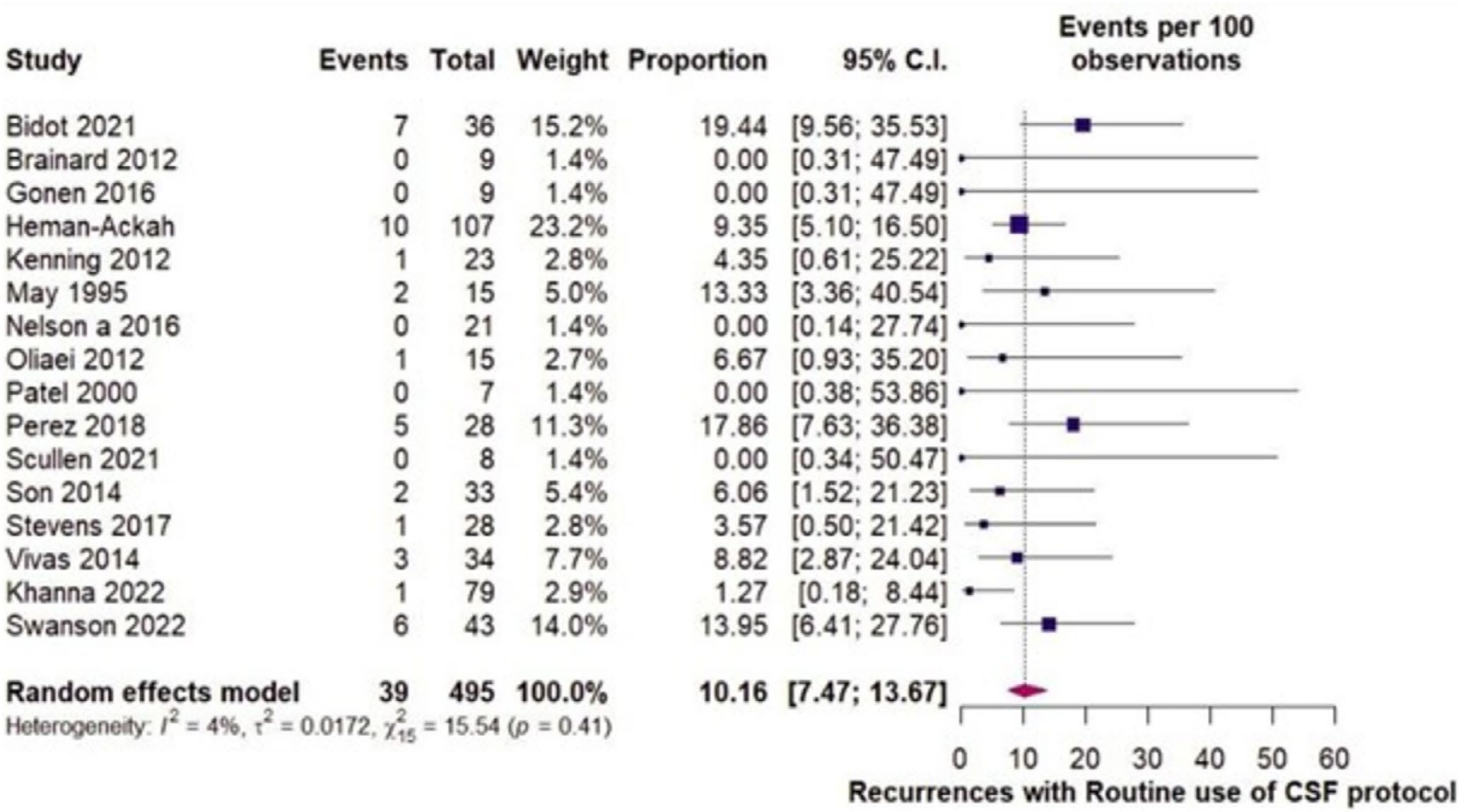
Forest plot demonstrating recurrence rates with routine or selected use of LP or LD.

**FIGURE 3:**
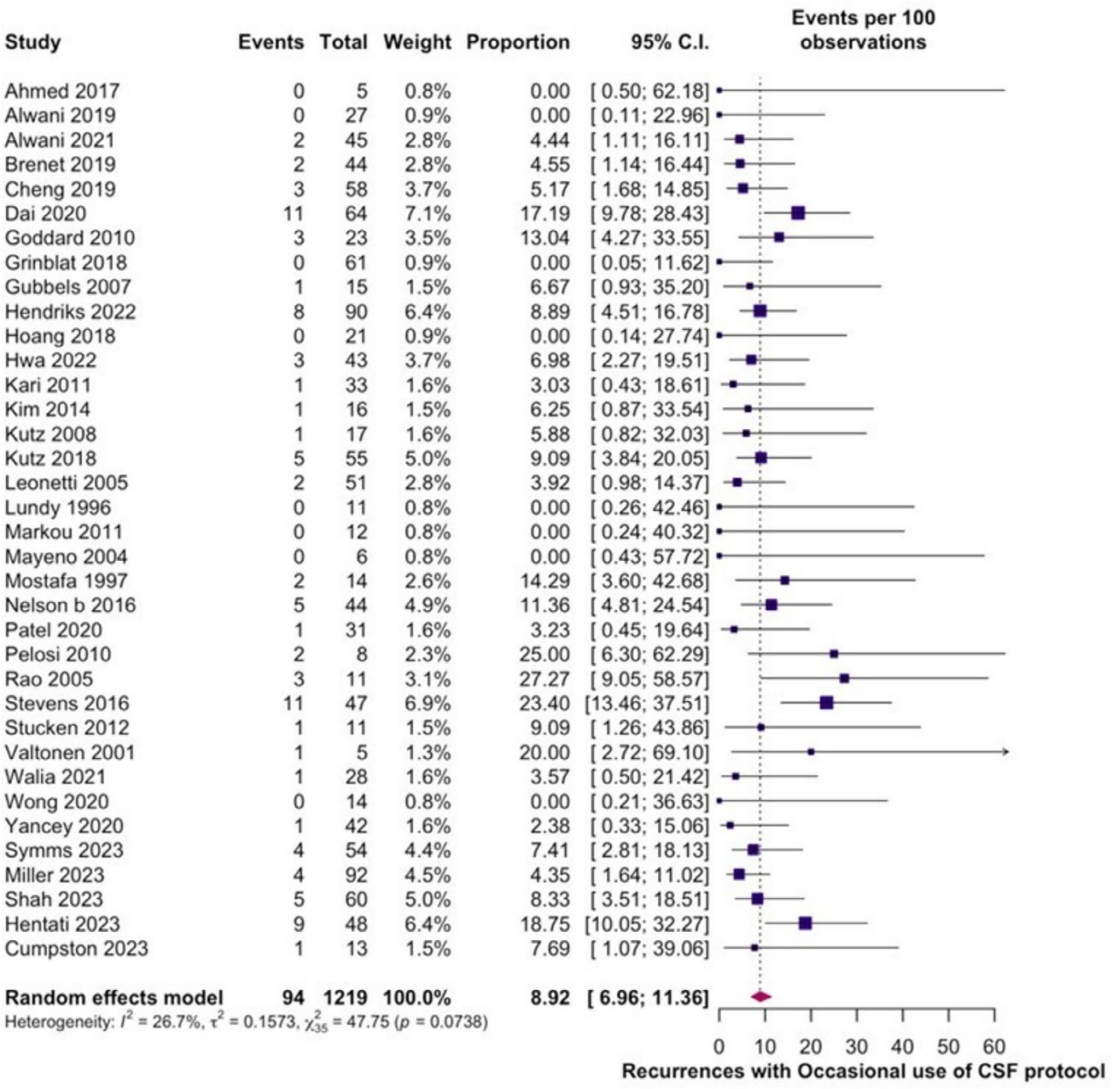
Forest plot depicting recurrence rates with occasional use of CSF protocol.

Under single arm proportional analysis, in 495 repairs (Figure 2) where LP/LD was used routinely or in selected patients, the recurrence or persistent rate of CSF leak was 10.16% (95% CI: 7.47%–13.67%; *I* ^2^ = 4%), with slight asymmetry in visual assessment of funnel plot (Figure S1) and rank correlation test negative for significance in asymmetry (*p* = 0.41, Figure 2). In 1219 repairs where LP/LD was utilized occasionally, the recurrence or persistent rate of CSF leak was 8.92% (95% CI: 6.96%–11.36%; *I* ^2^ = 26.7%) (Figure 3). To investigate heterogeneity, sensitivity analysis with a random effects model was performed, excluding one study at a time (Figure S2). Rates did not vary and were consistent with 9%, while Baujat plots were not suggestive of outliers or influential studies (Figures S3 and S4). Funnel plot (Figure S5) revealed slight asymmetry with Rank Correlation test negative for significance in asymmetry (*p* = 0.27).

Of the studies that used LP/LD as an option for postoperative recurrence, 54.4% of the patients responded well with leak resolution [7, 9, 22, 31, 40] (Table 3). Nelson's cohort [22] reported the most consistent success rate (100%). None of the studies reported any significant complication associated with LP or LD use.

**TABLE 3:** Studies where LP/LD was used as last option.

Study	Operations	Post‐op LP/LD	R/P	Revision	Success
Bidot et al. [7]	36	2	7	7	0
Cheng et al. [31]	58	2	3	2	N/a
Hwa et al. [40]	43	1	3	3	0
Kenning et al. [9]	23	1	1	0	1
Nelson et al. [22]^a^	44	5	5	0	5
		11	19	12	6 (54.5%)^b^

^a^ The second decade from [22].

^b^ Calculated for studies where LP/LD was used in patients with recurrence/persistence and success rate was either reported or able to calculate.

### 3.4 IIH

Across the reviewed studies, 10 reported diagnoses of IIH in patients with sCSFL. IIH criteria, protocol of investigation and prevalence were inconsistently reported. The prevalence of definite or presumed IIH was 14.3% (Table 4). Bidot et al. reported a prevalence of definite IIH, defined according to the 2013 revised Dandy criteria [65], of 19.4% in spontaneous CSF leak patients overall, increasing to 27.8% when presumed cases were included; the prevalence was lower (14%) in the subset with lateral skull base leaks [6]. Symms et al. [59] identified seven patients with IIH based on elevated OPs or papilledema, two of whom ultimately required VPS placement. Shah et al. found that 80% (four out of five) of patients with unsuccessful repairs were diagnosed with IIH, either before initial surgery or after failure of repair [15]. Hwa et al. reported only five patients who underwent neuro‐ophthalmological work‐up [40]. Neither Kutz et al. [6] nor Son et al. [25] assessed systematically for IIH, though isolated cases were noted.

**TABLE 4:** Studies with IIH guided practice.

Study	Operations	Protocol/method of confirmation	IIH findings
Bidot et al. [7]	36	2013 Dandy's criteria for IIH	7 patients (19.4%) had definite IIH, 3 (8.4%) had presumed. Of them anterior leaks were 8 and lateral 2
Hatch et al. [37]	18	History only	2 patients with IIH PMx
Hwa et al. [40]	5	Consultation with neuro‐ophthalmology	5 patients had pre‐op clinical assessment. None had IIH. None of them had recurrence/persistence
Kutz et al. [6]	17	n/a	2 patients with IIH. Both needed multiple craniotomies and eventually VPS
Son et al. [25]	33	n/a	2 patients had empty sella and high BMI (unconfirmed IIH)
Stevens et al. [65]	28	Headaches and blurred vision referred. Confirmed only by raised opening pressure	4 patients confirmed for IIH
Stevens et al. [14]	47	History only	7 patients with PMx IIH
Yancey et al. [2]	42	n/a	3 out of the 17 (17.6%) that underwent Lp had raised opening pressure, 17 (40.5%) had empty sella, 5 had IIH PMx (3/5 had raised opening pressure and 4/5 had empty sella). Retrospectively, only 6% would comply with definite IIH criteria
Symms et al. [59]	54	n/a	8 patients with IIH, 2 of them had VPS
Shah et al. [15]	55	N/a	10 (18.2%) patients diagnosed with BIH. Specifically, 3/5 of patients with bilateral defects had BIH, 3/5 patients with post‐op headaches and blurry vision had BIH and required VPS.
	335		48 (14.3%)

Abbreviations: LD, lumbar drain; LP, lumbar puncture; OP, opening pressure; PMx, past medical history.

Preoperative evaluation for increased OP was inconsistently performed across series. Data could be retrieved from five studies, but their quality did not allow for concrete analysis, other than descriptive analytics. At the very least, all five studies reported increased mean OP (Table 5). Vivas et al. [12] reported an 69% incidence of raised OP and a higher recurrence risk and VPS usage among them, a finding replicated by Khanna et al. [13], who found higher OP values in the group of patients that needed VPS.

**TABLE 5:** Studies without dedicated IIH protocol, but with IIH‐related opening pressure data.

Study	Operations	Protocol / Method of confirmation	IIH findings
Cooper et al. [26]	46	LD placed for 3 days peri‐op	No formal IIH assessment. Mean opening pressure was raised (MCF 21.7 ± 7.5; PCF 24 ± 3.6)
Kenning et al. [9]	23	LD placed for 3 days peri‐op	No formal IIH assessment. Mean opening pressure was raised (21.8 ± 6). Out of 10 patients with opening pressure > 20 (26.1 ± 3) VPS was placed in 4
Patel et al. [51]	31	Occasional use of LP. LD placed according to surgeon preference	No formal IIH assessment; 7 patients underwent LP. Mean opening pressure was raised (31 ± 5); 12 LD were placed
Vivas et al. [12]	34	Peri‐op LP or within 1 month post‐op	29 patients with OP measured. Median OP raised (29, range 13–36); 22/32 (69%) had OP > 20 cm H_2_O and 13 (40%) > 25 cm H_2_O. Median OP was higher in the shunt group (26 vs. 19, *p* < 0.05); 2/3 patients that needed revision surgery had raised OP > 20 cm H_2_O
Khanna et al. [13]	79	Peri‐op LD (2–3 days) and 2‐week follow‐up LP	Raised mean OP (21.8 ± 6). Higher OP in patients that needed VPS

Abbreviations: LD, lumbar drain; LP, lumbar puncture; MCF, middle cranial fossa; OP, opening pressure; PCF, posterior cranial fossa; PMx, past medical history.

### 3.5 Acetazolamide

In our review acetazolamide was only administered postoperatively. Nine studies addressed its use following lateral sCSFL repair (Table 6). Acetazolamide was administered in 78 out of 440 patients (17.7%). Only one study [19] administered acetazolamide routinely to all patients, reporting only one recurrence. Five studies used acetazolamide selectively in patients with definite or suspected IIH [5, 7, 14, 24, 32]. In these series, acetazolamide was administered in 13 out of 18 patients with known or newly diagnosed IIH (72.2%), but recurrence outcomes were not reported. In three studies acetazolamide was used for recurrent leaks in 57% (11 out of 19 recurrences received acetazolamide) [11, 15, 27].

**TABLE 6:** Use of acetazolamide after repair of lateral sCSFL.

Study	Operations	Patient population	Indication for acetazolamide	Dose/duration^a^	Outcomes^b^
Bidot et al. [7]	36	Definite/suspected IIH (*n* = 27)	IIH guided by neuro‐ophthalmic evaluation	Not specified	No recurrence data
Brainard et al. [24]	9	IIH (*n* = 6)	IIH with headache, visual changes, fundoscopic findings	Not specified	No recurrence data
Brenet et al. [11]	44	One recurrent leak (BMI 30 kg/m^2^) (*n* = 1)	Recurrence management	Not specified	Leak resolved, no recurrence at 37 months' follow‐up
Dai et al. [32]	64	Non‐iatrogenic leaks (*n* = 2)	IIH guided	Not specified	No recurrence data
Heman‐Ackah et al. [27]	107	Recurrence (*n* = 6) and other primary retrospectively retrieved (*n* = 10)	Recurrence	Not specified	No events reported
Kutz et al. [5]	55	Post‐CSF fistula with elevated ICP (*n* = 3)	IIH guided	Not specified	No recurrence data
Oliaei et al. [19]	15	All postoperative patients (*n* = 15)	Routine protocol	125 mg BID → 500 mg BID, 6 weeks	1 recurrence (patient did not receive standard repair material due to religious beliefs)
Stevens et al. [14]	47	IIH (*n* = 7)	IIH guided	Not specified	No events reported
Shah et al. [15]	60	Recurrent leaks (*n* = 5)	Recurrence management	Not specified	4/5 received acetazolamide prior to second surgery; individual outcomes not detailed
	440	78 (17.7%)			

^a^ BID = twice daily; dosage and duration not reported unless stated.

^b^ “No recurrence data” indicates that the study did not specify recurrence outcomes for patients receiving acetazolamide.

### 3.6 Shunts

Eighteen studies mentioned VPS usage (Table 7). As VPS related data were mixed with respect to defect location (anterior and lateral skull base) and in keeping with the fact that there is no study reasoning location related difference in this aspect, we included all VPS cases reported for sCSFL. VPS was used in 11.2% of patients, mostly for patients with high‐risk background or event of leak recurrence.

**TABLE 7:** Studies with VPS data.

Study	Operations	Shunts	Indication
Bidot et al. [7]	36	5	1 IIH, 1 recurrence, 3 unknown.
Kenning et al. [9]	23	6	IIH management or individual factors (high BMI, high volume leak)
Perez et al. [8]	28	2	1 obstructive hydrocephalus, 1 IIH (both after failed MCF repair)
Son et al. [25]	33	1	Recurrence
Vivas et al. [12]	34	16	Recurrence, pre‐existing IIH+ new CSF leak, OP > 25 cmH_2_O.
Khanna et al. [13]	79	13	11 IIH, 1 leak, 1 morbid BMI.
Alwani et al. [1]	45	1	IIH
Brenet et al. [11]	44	1	Recurrence
Dai et al. [32]	64	18	Recurrence
Kim et al. [10]	16	1	IIH
Kutz et al. [6]	17	2	IIH
Kutz et al. [5]	55	2	IIH
Schwartz et al. [3]	12	1	1 recurrence, 1 unknown.
Stevens et al. [14]	47	5	Recurrence
Yancey et al. [2]	42	3	Recurrence
Symms et al. [59]	54	1	IIH
Shah et al. [15]	60	3	IIH
Hwa et al. [40]	43	1	Recurrence
	732	82 (11.2%)	

Abbreviation: IIH, idiopathic intracranial hypertension.

## 4 Discussion

### 4.1 Perioperative Use of Lumbar Puncture and Drain

Routine perioperative LP/LD use can be justified by better surveillance of ICP [7, 9, 12, 13, 23, 26], intraoperative visualization of the defect thanks to brain relaxation [2, 15, 19, 23] and postoperative decrease of ICP and leakproof resolution of the bony defect at the site of repair where materials have been applied [7, 8, 9, 18, 22, 23, 24, 31, 32, 40, 46, 48, 52, 61, 64]. In addition, the placement of a LD enables intrathecal fluorescein dye injection, if needed, thus facilitating the intraoperative leak localization [50]. Dai et al. report, though in a mixed population of spontaneous and non‐spontaneous leaks, a resolution rate of up to 9.6%, in 8 out of 83 patients, just with the LD placement [32]. As falsely low ICP can be measured preoperatively in patients with active CSF leak or postoperatively in patients with unsuccessful repair, a more educated practice could be to guide the decision for further CSF diversion after obtaining ICP measurements both pre‐ and post‐ operatively [13], accounting for any false negative findings of a single ICP measurement. In our analysis (Tables 2 and 3), although we did not find any difference of recurrence or persistence rates between routine‐or‐selection and occasional groups, 54.5% of the patients with postoperative leak recurrence or persistence, avoided revision surgery after LD placement for persistent or recurrent leak. Currently there is not consistent evidence in the literature that could guide the selection of patients that would benefit from pre‐operative ICP surveillance.

Though it has been suggested that CSF leak is a symptom of increased ICP, bibliography shows that increased ICP is found only in 10%–35% [22]. Recent literature suggests that transiently increased ICP, as happens for example in the setting of OSA, may be equally significant in causing a leak through thinning of the bone [66] while still remaining undetected with LP or untransduced LD. Nelson et al. suggest that other causes of transient ICP elevation, eg. OSA, may play a more significant role and in keeping with this, preoperative LD does not affect success rate, while it increases length of stay (LOS) and costs [22]. It's role, they suggest, is therefore only to be used as a last resort option for unsuccessful surgeries [21]. Multiple studies argue that routine LD placement increases LOS and hospitalization costs [8, 46]. Moreover, false‐negative low preoperative OP measurements have been reported to cause delays in the decision for a definitive diversion treatment [13], and its postoperative use has been argued to hamper the early identification of inadequate defect repairs [43]. Oliaei and Patel [19, 23] advocate against LD entirely for TM approach, while seeing an indication only for MCF approach. This review did not find any significant LD‐associated complications.

Though it is established that neither single ICP measurements [67] nor clinical manifestations such as papilledema [68] are sufficiently reliable to make subsequent long‐term clinical decisions, there is a paucity of evidence in the use of more advanced investigation methods such as the transduced LD or ICP bolt, which have not been used in the management of sCSFL as they have been considered for other CSF abnormalities, such as hydrocephalous [69]. Recent advancements in non‐invasive ICP monitoring [70] and the introduction of machine learning models [71] may provide us with a non‐invasive alternative ICP measurement technique in the near future that could circumvent the variability of perioperative false measurements.

### 4.2 IIH

A formal diagnosis of IIH requires fulfillment of the modified Dandy criteria. However, the cutoff value for elevated ICP is controversial [72]. Why IIH is not always present in sCSFL patients but raised ICP is a risk factor for recurrence of persistence of sCSFL, are paradoxes that can be explained.

While classic neuro‐ophthalmic signs of intracranial hypertension, such as papilledema or cranial nerve VI palsy, are uncommon in patients without a prior history of IIH, patients may still harbor chronically elevated ICP that drives progressive bone thinning and sCSFL [73, 74]. However, it's prevalence in sCSFL patients is less than 15% in the bibliography [7, 40], as was in our review (15.4%). The phenomenon of the leak itself acting as a “pop‐off valve” may explain why some patients lack overt IIH symptoms at presentation. Brainard et al. investigated this theory and found increased ICP postoperatively in six out of nine (67%) sCSF leak patients, while three of them asymptomatic [24]. Similarly, Kutz et al. found increased ICP in five out of 14 (35.7%) patients, 6 weeks postoperatively [5].

Studies have shown that untreated or borderline elevated ICP is associated with an increased risk of recurrence after repair [12, 59]. Prevalence of IIH is significantly higher in revision cases when compared to primary repairs (41.7% vs. 13.7%, *p* = 0.015) [27]. Several studies, including Stevens et al. [14, 17] and Vivas et al. [12], documented a shift in practice toward preoperative assessment of ICP and the use of CSF diversion or acetazolamide in select patients—typically those with known IIH, borderline ICP, or recurrence after initial repair. These data highlight the growing appreciation for the role of raised ICP.

The evidence underscores a critical need for systematic preoperative assessment of IIH in patients presenting with lateral sCSFL, which includes fundoscopy, visual field testing, measurement of OP, and MRI/MRV for radiographic markers of raised ICP (e.g., empty sella, cephaloceles, optic nerve sheath dilation, transverse sinus stenosis) [7, 12, 15].

### 4.3 Acetazolamide

Acetazolamide is widely used in IIH patients and as an adjunctive measure to improve outcomes after sCSFL repair. A recent systematic review by Raub reported that adjunctive acetazolamide increased the success rate of surgical repair in patients with postoperative intracranial hypertension after repair of anterior cCSF leak, and in some cases, acetazolamide alone resolved leaks without the need for surgery [75]. Oliaei et al. reported favorable healing with routine postoperative acetazolamide mixed skull base populations [19], while Teachey et al. showed higher success rates with use of acetazolamide in patients with raised ICP [76]. Specifically, in the lateral skull base population, evidence is more limited. Available studies suggest postoperative use of acetazolamide in patients with confirmed or suspected IIH [7, 77] or persistent leaks [11]. In one series, postoperative acetazolamide was prescribed in up to 50% of revision patients, with no documented second recurrences in that cohort [27].

Overall, the limited but consistent evidence [75, 76] suggests that adjunctive acetazolamide may be beneficial in recurrent leaks, documented IIH, or patients at high risk for elevated ICP. Optimal dosing, duration, and patient selection remain undefined for lateral skull base surgery.

### 4.4 Shunts

The role of VPS has been variably reported, with no clearly defined indications for its pre‐ and post‐operative use. Firstly, VPS can be used in the setting of increased ICP. In Shah et al. [15] 18.2% exhibited signs of IIH and 5.4% required VPS. Symms et al. [59] found that refractory IIH is more determinative of failure than graft material. In Vivas et al. [12] 53% of patients underwent CSF diversion, mostly via VPS. Patients selected for shunting had significantly higher ICPs (median 26.2 vs. 19.6 cm H_2_O) and BMI (37 vs. 33 kg/m^2^). Similarly, Khanna et al. [13] reported higher ICP values for patients shunted during first admission (26.2 vs. 20.4 cm H_2_O). Secondly, VPS is employed following failed primary repair. Stevens et al. [14] noted that 10.4% of patients with IIH who developed recurrence, underwent VPS placement. Likewise, Schwartz et al. [3] described two such cases. The experience of In Alwani et al. [1], a patient with delayed postoperative rhinorrhea had elevated ICP and subsequently underwent VPS placement, with no further interventions needed thereafter.

While VPS is accepted in refractory cases, it's potential benefit as a primary prophylactic measure remains debated [2, 15].

## 5 Conclusion

In conclusion, although LP and LD use is usually safe and feasible, it is not always justified as perioperative management option in sCSFL. It is an invasive procedure which can lead to potential morbidity and increase in LOS and hospitalization costs, as potentially precipitate the inappropriate use of permanent CSF diversion. Patient selection should be considered with these potential risks in mind. Although most data are derived from anterior skull base series, the principle of assessing and addressing IIH likely extends to lateral skull base repairs. Ultimately, incorporating standardized preoperative evaluation and targeted management of IIH may improve long‐term repair durability and reduce recurrence in this challenging patient population. Surveillance with routine follow‐up for new‐onset postoperative IIH identification should be considered. Acetazolamide and VPS are both options for the management of postoperative recurrence or IIH symptoms. Individualized assessment, incorporating ICP measurements, BMI, radiologic findings and clinical surveillance is crucial in deciding between medical or surgical ICP management. We suggest that more prospective studies should be carried out, to investigate the role of LD, acetazolamide and VPS in the perioperative management of sCSFL.

## Conflicts of Interest

The authors declare no conflicts of interest.

## Supporting information


**Figure S1:** Funnel plot analysis for recurrences in Routine group.
**Figure S2:** Leave One Out sensitivity analysis for recurrences in Occasional group.
**Figure S3:** Baujat plot for recurrences in Occasional group.
**Figure S4:** Influential studies for recurrences in Occasional group.
**Figure S5:** Funnel plot analysis for recurrences in Occasional group.

## Data Availability

The data that support the findings of this study are available from the corresponding author upon reasonable request.
