# Supplementary material for: Spontaneous Middle Cranial Fossa Cerebrospinal Fluid Leaks and Intracranial Hypertension: Systematic Review With Meta‐Analysis
Source: Laryngoscope Investig Otolaryngol. 2026 May 12;11(3):e70439. doi: 10.1002/lio2.70439 (PMC13168526; doi:10.1002/lio2.70439)

**Supplemental Materials**

Figure S1. Funnel plot analysis for recurrences in Routine group
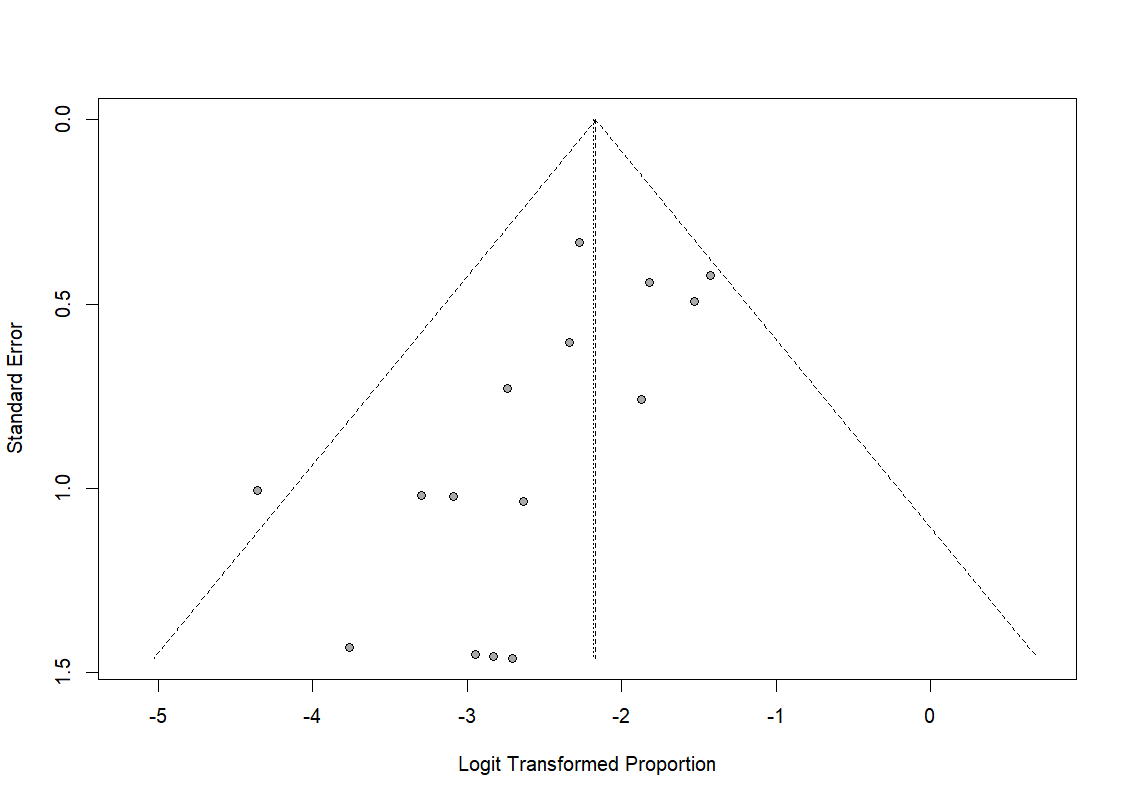
Figure S2. Leave One Out sensitivity analysis for recurrences in Occasional group


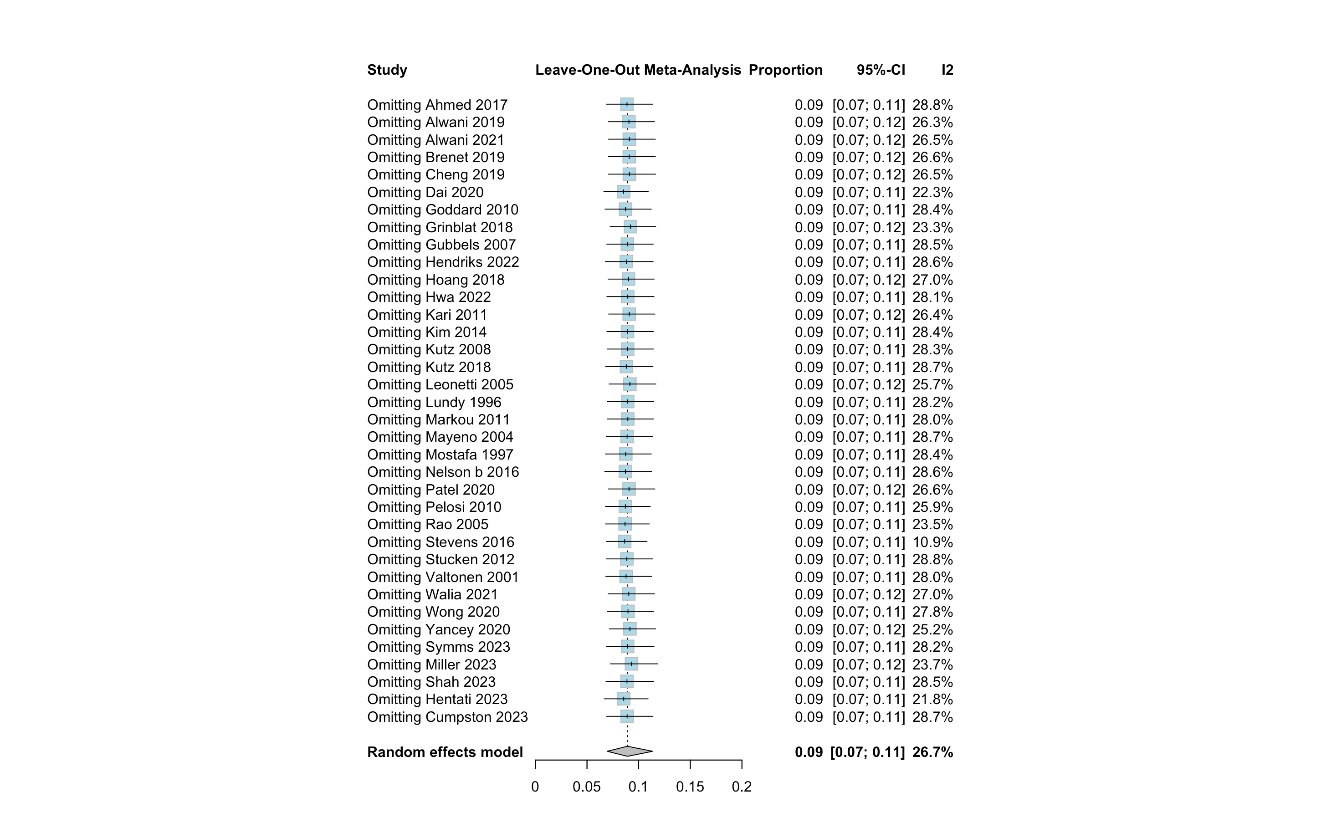


Figure S3. Baujat plot for recurrences in Occasional group


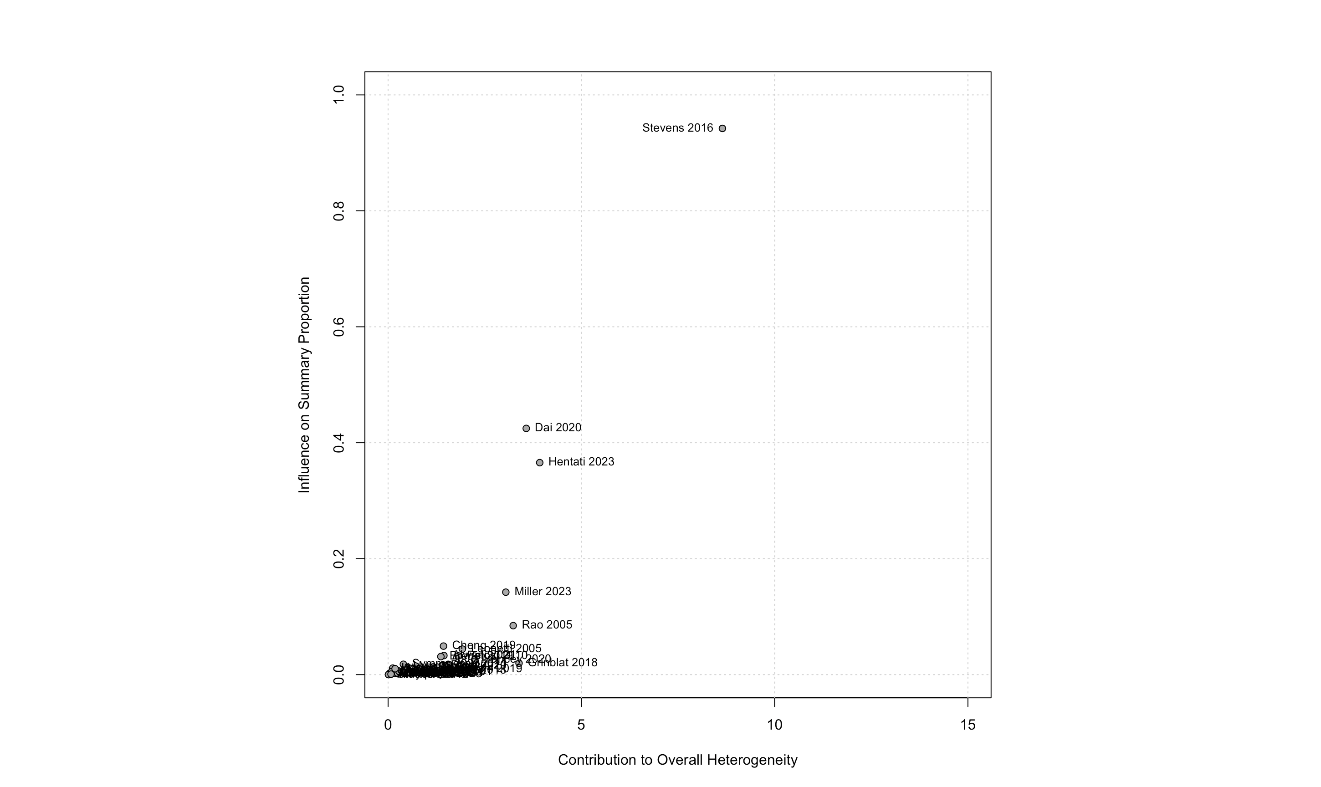


Figure S4. Influential studies for recurrences in Occasional group


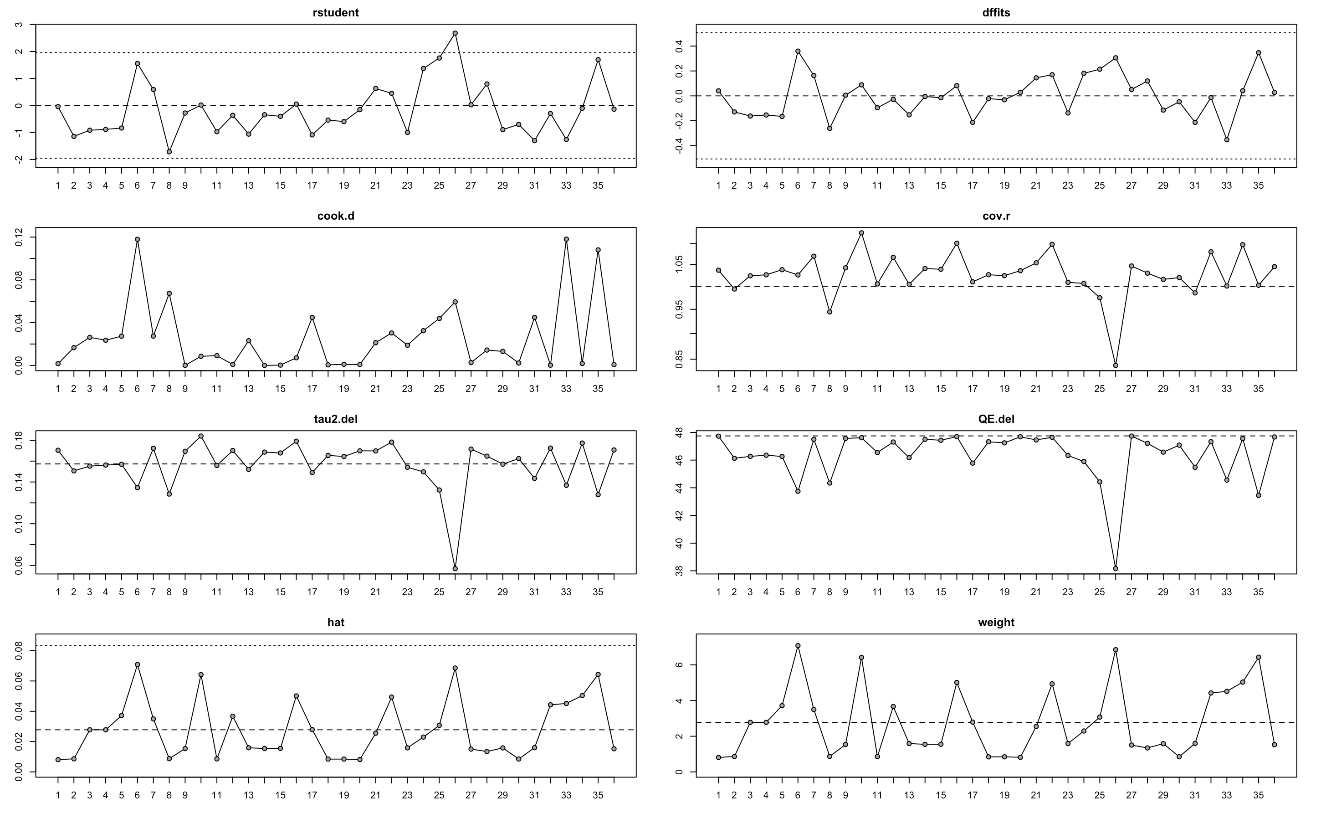


Figure S5. Funnel plot analysis for recurrences in Occasional group


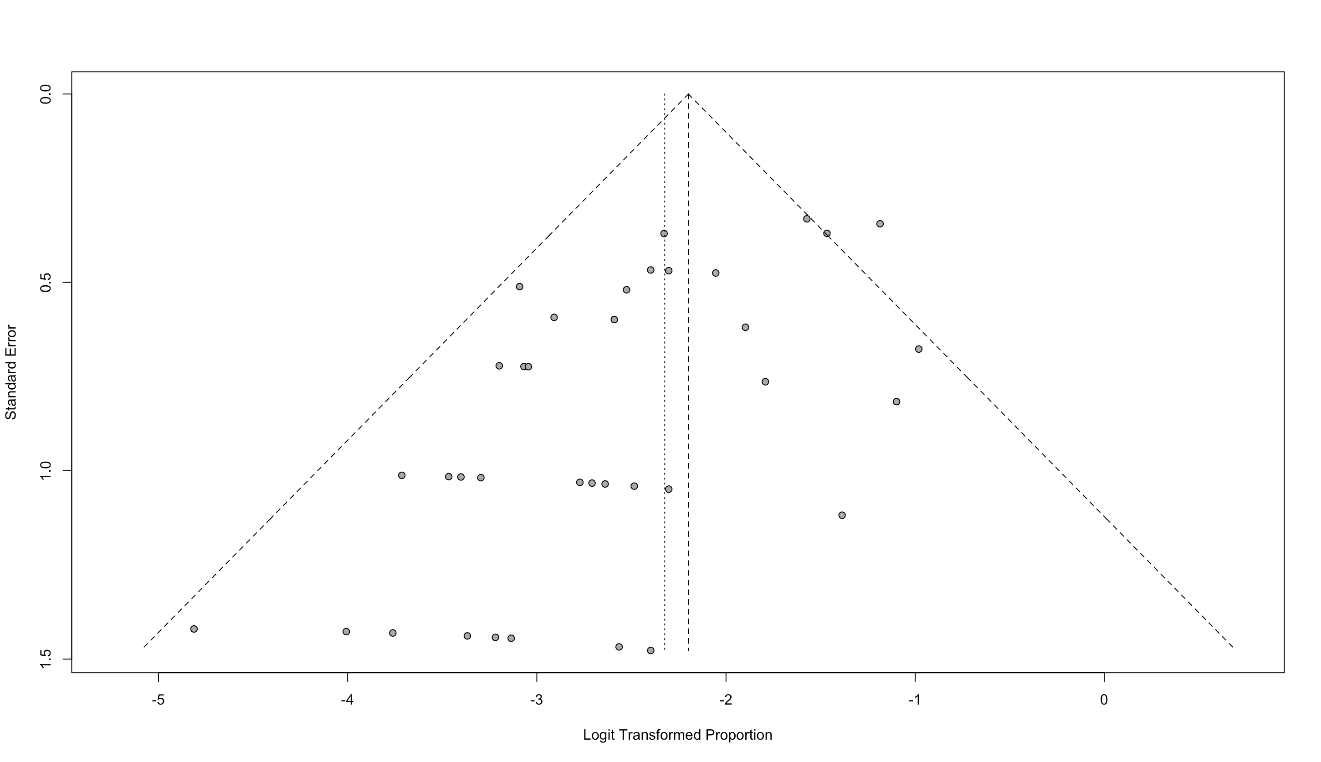

Supplement: Supplementary file 1 — Figure S1: Funnel plot analysis for recurrences in Routine group. Figure S2: Leave One Out sensitivity analysis for recurrences in Occasional group. Figure S3: Baujat plot for recurrences in Occasional group. Figure S4: Influential studies for recurrences in Occasional group. Figure S5: Funnel plot analysis for recurrences in Occasional group. [file LIO2-11-e70439-s001.docx]
